# An impedimetric determination of alkaline phosphatase activity based on the oxidation reaction mediated by Cu^2+^ bound to poly-thymine DNA†

**DOI:** 10.1039/c7ra13642k

**Published:** 2018-03-21

**Authors:** Joon Young Lee, Jun Ki Ahn, Ki Soo Park, Hyun Gyu Park

**Affiliations:** Department of Chemical and Biomolecular Engineering (BK 21+ Program), KAIST Daehak-ro 291, Yuseong-gu Daejeon 305-338 Republic of Korea hgpark@kaist.ac.kr +82-42-350-3910 +82-42-350-3932; Department of Biological Engineering, College of Engineering, Konkuk University Seoul 05029 Republic of Korea

## Abstract

We herein describe a novel impedimetric method to determine alkaline phosphatase (ALP) activity based on the Cu^2+^-mediated oxidation of ascorbic acid on a specific DNA probe-modified electrode. In this method, pyrophosphate (PPi) capable of complexing with Cu^2+^ is employed as a substrate of the ALP enzyme. In the presence of ALP, PPi is hydrolyzed to phosphate (Pi), which is not able to entrap Cu^2+^. The free Cu^2+^ are specifically bound to a poly-thymine DNA probe immobilized on the electrode surface and reduced to form copper nanoparticles by a concomitant oxidation of ascorbic acid. As a result, the oxidation products of ascorbic acid are accumulated on the electrode surface, which consequently increase electron transfer resistance (*R*_et_) by interrupting the electron transfer on the electrode. On the other hand, in the absence of ALP, PPi remains intact to effectively capture Cu^2+^, consequently preventing the oxidation of ascorbic acid and the subsequent increase of *R*_et_. Based on this design principle, the change in *R*_et_, which is proportional to ALP activity, was measured by electrochemical impedance spectroscopy (EIS) and ALP activities were successfully determined down to 6.5 pM (7.2 U L^−1^) with excellent selectivity.

## Abstract 2

A novel impedimetric assay for the accurate determination of alkaline phosphatase (ALP) activity is developed based on the Cu^2+^-mediated oxidation of ascorbic acid on a poly-thymine DNA-modified electrode.

## Graphical abstract

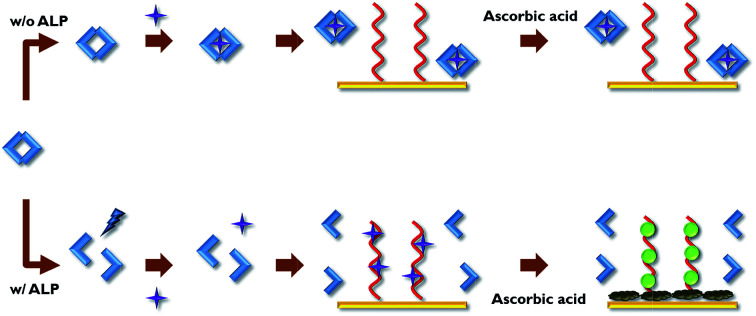

## Introduction

Alkaline phosphatase (ALP) is an enzyme that catalyzes the hydrolysis and transphosphorylation of a wide variety of phosphoric acid monoesters. The enzyme plays a vital role in the regulation of several biologically important intracellular processes associated with cell cycle, growth, apoptosis and signal transduction pathways. Therefore, the concentration of ALP in the human body needs to be routinely monitored as a diagnostic indicator of various diseases including breast cancer, liver disease, bone disease, diabetes, hepatitis, and kidney cancer.^1–3^

The representative commercial kits for ALP assay employ *p*-nitrophenyl phosphate, 4-methylumbelliferyl phosphate and *p*-aminophenyl phosphate. ALP hydrolyzes these substrates into *p*-nitrophenol, 4-methylumbelliferone and *p*-aminophenyl, which are yellow colored, fluorescent and electroactive products, respectively.^4–8^ Besides, many other methods based on chemiluminescence,^9^ fluorescence,^10–14^ surface Raman scattering,^15,16^ and electrochemistry^17–22^ have been intensively developed to determine the ALP activity. Among these methods, the electrochemical methods, with several advantageous characteristics including their portability and cost-effectiveness, have great promise for low-cost miniaturized easy-to-use portable devices for various chemical and biological applications.^23–30^ For instance, Miao *et al.* and Zhang *et al.* designed exonuclease-mediated signal amplification methods for a sensitive, electrochemical ALP assay, respectively. The systems take advantage of recycling of DNA probe that serves as a template for exonuclease, which regulates the accessibility of redox probes toward electrodes and consequently enables the determination of a very low amount of ALP. However, they still have several limitations such as complex synthesis of ALP substrates and complicated processes that involve multiple enzymes.^19,20^ Therefore, it is still highly demanded to develop more cost-effective and convenient electrochemical method for the assay of ALP activity.

Recently, Qing *et al.* described an ascorbic acid-catalyzed reduction of Cu^2+^ entrapped within poly-thymine DNA probe for the formation of fluorescence copper nanoparticle (CuNP), which has been used for development of various bioassays.^31–38^ Taking this unique feature, Ocaña *et al.* developed an impedimetric method for sensing Cu^2+^.^39^ In this method, the rate of ascorbic acid oxidation was significantly enhanced by DNA-templated Cu^2+^ on the electrode,^40,41^ and consequently the electrode surface was covered with oxidation products, resulting in an increase in the electron transfer resistance (*R*_et_) of the electrode. The change in the *R*_et_ is monitored by measuring electrochemical impedance (EIS) spectroscopy.

In this study, we designed a new electrochemical system to determine ALP activity, which has following features. First, the redox reaction of Cu^2+^ bound to poly-thymine DNA probe and ascorbic acid is utilized to produce a precipitated oxidation product. Next, the capability of Cu^2+^ to mediate oxidation reactions of ascorbic acid is remarkably inhibited by its complexation with PPi. The final factor of the proposed detection system is that inhibition of Cu^2+^-mediated oxidation of ascorbic acid is eliminated by ALP activity, which hydrolytically cleaves PPi to form Pi. By employing this design principle, we successfully determined the ALP activity with excellent selectivity and verified the clinical capability by detecting ALP in human blood serum.

## Experimental

### Materials

Thiol-modified DNA probe (5′-thiol-T_20_-3′) was synthesized and purified by high-performance liquid chromatography (HPLC) by Integrated DNA Technologies (Coralville, USA). Alkaline phosphatase (ALP), sodium pyrophosphate (PPi), copper chloride (CuCl_2_), tris(hydroxymethyl)aminomethane, potassium chloride (KCl), sodium chloride (NaCl), ascorbic acid, 3-morpholinopropane-1-sulfonic acid (MOPS), potassium ferricyanide (K_3_[Fe(CN)_6_]), potassium ferrocyanide (K_4_[Fe(CN)_6_]), lysozyme, albumin, avidin, glucose oxidase, adenosine 5′-triphosphate (ATP), creatinine, cysteine, glucose and human serum were purchased from Sigma-Aldrich (St. Louis, USA). Ultrapure DNase/RNase-free distilled water (DW) was purchased from Bioneer® (Daejeon, Korea).

### Preparation of DNA probe-modified electrode

Titanium (20 nm) was first coated on Si wafer, followed by 200 nm gold (99.9%) thin layer formation by using an e-beam evaporator. The gold electrode was cleaned with piranha solution (H_2_SO_4_ : H_2_O_2_ = 4 : 1) for 10 min and thoroughly washed with phosphate buffered saline (PBS, 50 mM sodium phosphate, 75 mM sodium chloride, pH 7.2).^42^ The gold electrode was then treated with 200 μL of an aqueous solution containing 1 μM thiol-modified poly-thymine DNA probe (5′-thiol-T_20_-3′) for 2 h. The DNA-modified electrode was washed with PBS and treated with 1 mM mercaptohexanol solution for 20 min to block bare gold surface, which is not covered by the thiol-modified DNA probe. Finally, the DNA-modified electrode was washed with PBS and water.

### Confirmation of the effects of PPi on the reduction of Cu^2+^

38 μL of PPi solutions at varying concentrations, 5 μL of 1 mM Cu^2+^, 1 μL of 100 mM ascorbic acid, 1 μL of 100 μM poly-thymine DNA probe (5′-T_20_-3′) and 5 μL of 100 mM MOPS were mixed to make reaction solution of 50 μL in 10 mM Tris–acetate (pH 7.4), which was then incubated at room temperature for 3 min. The fluorescence signal of the formed CuNP were next measured at excitation wavelength and emission wavelength of 340 nm and 660 nm, respectively using a Tecan Infinite M200 microplate reader (Mannedorf, Switzerland).

### Procedure for ALP detection

10 μL of solution containing ALP at varying concentrations or lysozyme, albumin, avidin, and glucose oxidase at 100 nM were mixed with 10 μL of 1 mM PPi to prepare ALP reaction mixtures of 90 μL in 10 mM Tris–acetate (pH 7.4), which were then incubated at 37 °C for 60 min. Next, 10 μL of 1 mM CuCl_2_ was added to the ALP reaction products, followed by the incubation at room temperature for 20 min. The mixed solution was then applied to the DNA probe-immobilized electrode together with 2 μL of 100 mM ascorbic acid and 10 μL of 100 mM MOPS. After incubation at room temperature for 3 min, 1 mL of 4 mM [Fe(CN)_6_]^3−/4−^ was applied as a redox probe on the electrode and the impedance of the electrode surface was measured.

### ALP activity assay in human serum

10 μL of ALP solutions at varying concentrations was spiked into the solution of human serum diluted with 10 mM Tris–acetate (pH 7.4), which were subjected to the same procedures described above. For the determination of the ALP activity, the calibration curve was first carried out in human serum. The *R*_et_ value from the unknown samples was then obtained and analyzed based on the calibration curve.^43^

### Electrochemical measurement

A conventional three-electrode cell was used for the electrochemical measurement. The gold matrix electrode was used as a working electrode with Ag/AgCl reference electrode and platinum counter electrode. Electrochemical impedance spectroscopy (EIS) was performed using a GAMRY Reference 600 (Warminster, USA). Impedance was measured at an alternating voltage of 10 mV in the frequency range from 1 Hz to 100 kHz. Impedance spectra were recorded in the form of complex plane diagrams (Nyquist plots), and the experimental impedance data were analyzed by software, GAMRY eChem analyst (Warminster, USA).^44^

## Results and discussion

### The overall detection procedure

**Scheme 1:**
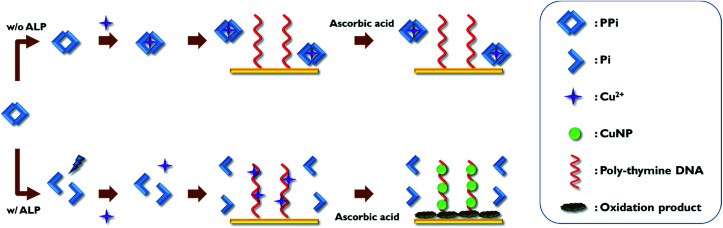
Schematic illustration of the electrochemical ALP assay utilizing Cu^2+^-mediated oxidation of ascorbic acid on the poly-thymine DNA-modified electrode.

The overall scheme of the electrochemical ALP detection method is illustrated in Scheme 1 in which Cu^2+^, specifically bound to poly-thymine DNA probe to mediate the oxidation of ascorbic acid, is used as a key detection component. To construct the detection system in this study, the thiol-modified poly-thymine DNA probe (5′-thiol-T_20_-3′) is first immobilized onto gold electrode. The modified electrode was then treated with mercaptohexanol solution to block bare gold surface. Thus, the non-specifically adsorbed ssDNA molecules were displaced and the DNA molecules on the surface of the electrode were erected, making it easier to mediate the oxidation of ascorbic acid.^45–47^ The assay begins by incubating a sample containing ALP with a PPi solution, which is then incubated with Cu^2+^. The resulting solution is applied to the poly-thymine DNA-modified electrode, which is subsequently incubated with ascorbic acid and MOPS solution. In the presence of ALP, PPi is hydrolyzed to Pi, preventing its complexation with Cu^2+^. Thus, the free Cu^2+^ are specifically entrapped by the poly-thymine DNA probe immobilized on the electrode where Cu^2+^ are reduced to form CuNP with the concomitant oxidation of ascorbic acid. As a result, the oxidation products are accumulated on the electrode, which interrupts the electron transfer on the electrode, thereby increasing the electron transfer resistance (*R*_et_). On the other hand, in the absence of ALP, PPi is retained intact and entraps Cu^2+^, consequently preventing the reduction of Cu^2+^ by the oxidation of ascorbic acid. Therefore, the oxidation products are not formed and there is no change observed for the electron transfer on the electrode. Based on this design principle, the change of the *R*_et_, which is proportional to ALP activity, is analyzed by measuring electrochemical impedance spectroscopy (EIS) and used to determine the ALP activity in a sample.

### Feasibility of the electrochemical ALP assay

The effects of PPi and ALP on the Cu^2+^-mediated oxidation of ascorbic acid were investigated by measuring the fluorescence signals arising from CuNP formed by reduction of Cu^2+^. As shown in Fig. S1(a),† fluorescent intensities decreased with increasing concentrations of PPi. These observations demonstrate that free Cu^2+^ are effectively reduced to form CuNP upon the application of ascorbic acid and the degree of Cu^2+^ reduction is suppressed by its complexation with PPi. The fluorescent signal from CuNP was almost completely diminished over 100 μM of PPi concentration, which was chosen as the optimal concentration of PPi to block the reduction of Cu^2+^. In addition, the effect of ALP activity on the fluorescence signal by Cu^2+^ reduction was also examined. The results in Fig. S1(b)† show that ALP present in the test sample, effectively recovered the diminished fluorescent signal, which confirms that ALP hydrolyzes PPi into Pi, preventing its complexation with Cu^2+^.

**Fig. 1:**
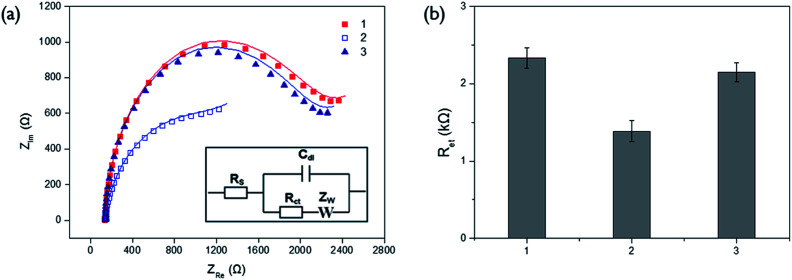
Electrochemical responses obtained from the Cu^2+^-mediated oxidation of ascorbic acid on the poly-thymine DNA-modified electrode. (a) Nyquist plots of the impedance spectra (inset: equivalent circuit for the impedance spectroscopy) and (b) electron transfer resistance (*R*_et_) obtained from the corresponding impedance spectra of solutions containing free Cu^2+^ and ascorbic acid (1), Cu^2+^ and ascorbic acid together with 100 μM PPi (2) or 100 μM PPi treated with 1 nM ALP (3). The final concentrations of Cu^2+^ and ascorbic acid are 1 μM and 2 mM, respectively.

**Fig. 2:**
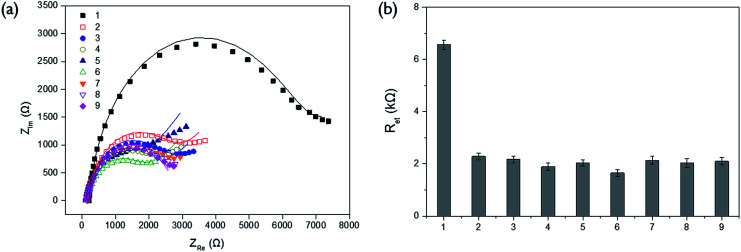
Specificity of the impedimetric method for ALP assay based on Cu^2+^-mediated oxidation of ascorbic acid on the poly-thymine DNA-modified electrode. (a) Nyquist plots of the impedance spectra and (b) electron transfer resistance (*R*_et_) obtained from the corresponding impedance spectra upon Cu^2+^-mediated oxidation in the presence of 1 nM ALP (1) and 10 nM of other proteins such as lysozyme (2), albumin (3), avidin (4), and glucose oxidase (5), and other biological molecules such as ATP (6), creatinine (7), cysteine (8), and glucose (9).

**Fig. 3:**
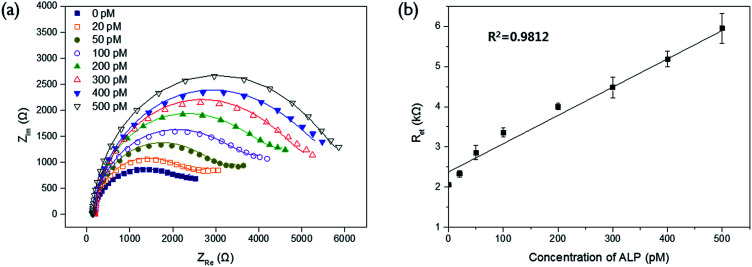
Sensitivity of the impedimetric method for ALP assay based on Cu^2+^-mediated oxidation of ascorbic acid on the poly-thymine DNA-modified electrode. (a) Nyquist plots of the impedance spectra and (b) electron transfer resistance (*R*_et_) obtained from the corresponding impedance spectra upon Cu^2+^-mediated oxidation in the presence of varying concentrations of ALP.

We next verified the feasibility of the proposed electrochemical ALP assay system by measuring the impedance spectra from different samples on the poly-thymine DNA-modified electrode (Fig. 1). In the presence of both Cu^2+^ and ascorbic acid, Cu^2+^ were reduced to form CuNP, concomitantly generating oxidation products of ascorbic acid, which were precipitated on the electrode, consequently resulting in the increase of *R*_et_ (Fig. 1a-1). However, PPi effectively suppressed the Cu^2+^-mediated oxidation of ascorbic acid by binding to Cu^2+^ and, as a result, *R*_et_ of the electrode decreased (Fig. 1a-2). Most importantly, when PPi was incubated with ALP, impedance signal produced by Cu^2+^-mediated oxidation was recovered almost comparable to that in the presence of both Cu^2+^ and ascorbic acid (Fig. 1a-1 and 3). These observations clearly indicate that ALP-catalyzed hydrolysis of PPi regulates the Cu^2+^-mediated oxidation.

The optimal reaction conditions for the efficient ALP activity assay were also determined by examining the *R*_et_ at different conditions. The results of experiments in which the reaction times for ALP reaction and Cu^2+^-mediated oxidation were varied, confirm that 60 min of ALP reaction and 3 min of Cu^2+^-mediated oxidation are ideal for further experiments (Fig. S2 and S3†).

### Selectivity and sensitivity of the ALP assay

In order to verify the selectivity of this assay system, the *R*_et_ values were examined for other proteins (lysozyme, albumin, avidin, and glucose oxidase) and biological molecules (ATP, creatinine, cysteine, and glucose) which are generally present in blood sample. As shown in Fig. 2, there was no significant enhancement of *R*_et_ observed from other proteins and biological molecules even at the ten times higher concentration than that of ALP while ALP effectively resulted in quite high level of *R*_et_. It indicates that only ALP is capable of hydrolyzing PPi, which consequently prevents its complexation with Cu^2+^ and increases the impedance signal. This result confirms high selectivity of our system for ALP assay.

The detection sensitivity of the proposed biosensor was next determined by measuring *R*_et_ with varying concentration of ALP under the optimal experimental conditions. The results in Fig. 3 show that the measured *R*_et_ increases with increasing concentration of ALP. An excellent linear relationship ((electron transfer resistance) = 0.0073 × (concentration of ALP) + 2.3436, *R*^2^ = 0.9812) was obtained in the range from 20 to 500 pM and the limit of detection (LOD) (3*σ*/slope, where *σ* is the standard deviation of blank results) was 6.5 pM (7.2 U L^−1^), which is comparable to those from other electrochemical methods for ALP detection (Table S1†).

### ALP assay in human serum

The practical applicability of the developed strategy was verified by determining the ALP activity in human serum. As presented in Fig. S4,† the impedance response to ALP in 1% human serum showed almost same tendency with the artificial samples containing only ALP, showing the linear relationship ((electron transfer resistance) = 0.0198 × (concentration of ALP) + 7.5011, *R*^2^ = 0.9825) in the range from 50 to 500 pM (Fig. S4†). The detection strategy exhibited excellent reproducibility and precision, as evidenced by the coefficients of variation (≤8.16%) and recovery rates (96.7–105.6%). These results clearly confirm that the present system has a potential to reliably analyze the activity of ALP in clinical samples (Table 1).

**Table 1:** Determination of ALP spiked in human serum samples

Added ALP (pM)	Measured ALP^a^ (pM)	SD^b^	CV^c^ (%)	Recovery^d^ (%)
150	147.0	12.00	8.16	98.0
250	264.1	10.12	3.83	105.6
350	338.6	13.88	4.10	96.7

a Mean of three measurements.

b Standard deviation of three measurements.

c Coefficient of variation = SD/mean × 100.

d Measured value/added value × 100.

## Conclusions

In this study, we successfully developed a novel electrochemical method for the determination of ALP activity by utilizing the Cu^2+^-mediated oxidation of ascorbic acid. Our ALP assay system basically relies on the impedance signal regulated by the PPi capable of complexing with Cu^2+^. The present strategy with a signal-on electrochemical response sensitively determined the ALP activity down to 6.5 pM (7.2 U L^−1^) with the high selectivity. In addition, its practical applicability was successfully demonstrated by determining the ALP activity in human serum. Importantly, the proposed electrochemical biosensor, which is relatively small and inexpensive compared to the conventional fluorescence instruments, enables the determination of ALP activity in a miniaturized and cost-effective ALP assay system. This study proves the successful application of the Cu^2+^-mediated oxidation for the electrochemical determination of ALP activity, which will lead to the further development of various enzyme activity assays.

## Conflicts of interest

There are no conflicts to declare.

## Supplementary Material

RA-008-C7RA13642K-s001
