# Supplementary material for: An impedimetric determination of alkaline phosphatase activity based on the oxidation reaction mediated by Cu2+ bound to poly-thymine DNA
Source: RSC Adv. 2018 Mar 21;8(20):11241–6. doi: 10.1039/c7ra13642k (PMC9078965; doi:10.1039/c7ra13642k)
Supplement: RA-008-C7RA13642K-s001 [file RA-008-C7RA13642K-s001.pdf]

Electronic Supplementary Material (ESI) for RSC Advances.
This journal is © The Royal Society of Chemistry 2018

Supplementary Information

An impedimetric determination of alkaline phosphatase activity based on the oxidation reaction mediated by Cu^{2+} bound to poly-thymine DNA

Joon Young Lee,[a] Jun Ki Ahn,[a] Ki Soo Park[b] and Hyun Gyu Park[a]*

[a] Department of Chemical and Biomolecular Engineering (BK21+ program), Korea Advanced Institute of Science and Technology (KAIST), 291, Daehak-ro, Yuseong-gu, Daejeon 305-701, Republic of Korea

[b] Department of Biological Engineering, College of Engineering, Konkuk University, Seoul 05029, Republic of Korea

*Corresponding author. Tel.: +82 42 350 3932; Fax: +82 42 350 3910; E-mail: hgpark@kaist.ac.kr

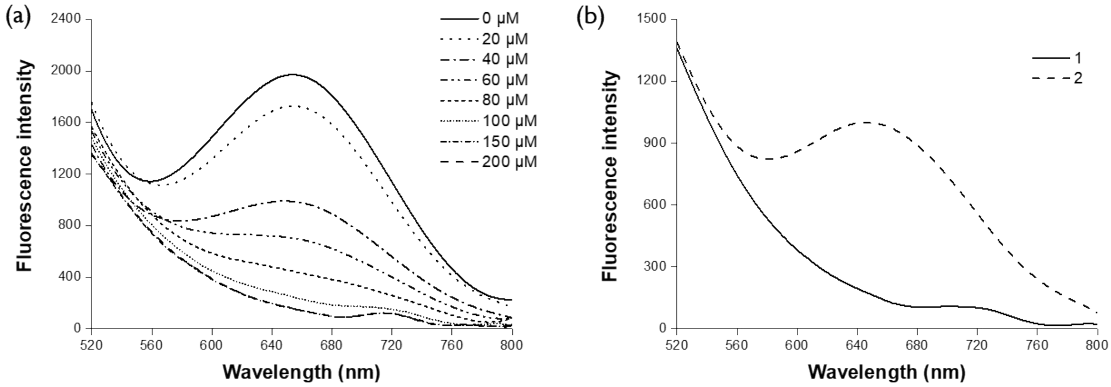

Fig. S1 Fluorescence spectra obtained from CuNP formed by the reduction of Cu^{2+}. (a) Fluorescence spectra from CuNP formed through treating Cu^{2+} with ascorbic acid in the presence of PPi at varying concentrations. (b) Fluorescence spectra obtained from solutions containing 100 µM Cu^{2+}, 2 mM ascorbic acid, 1 µM poly-thymine DNA probe and 100 µM PPi (1), or 100 µM PPi previously treated with 1 nM ALP (2).

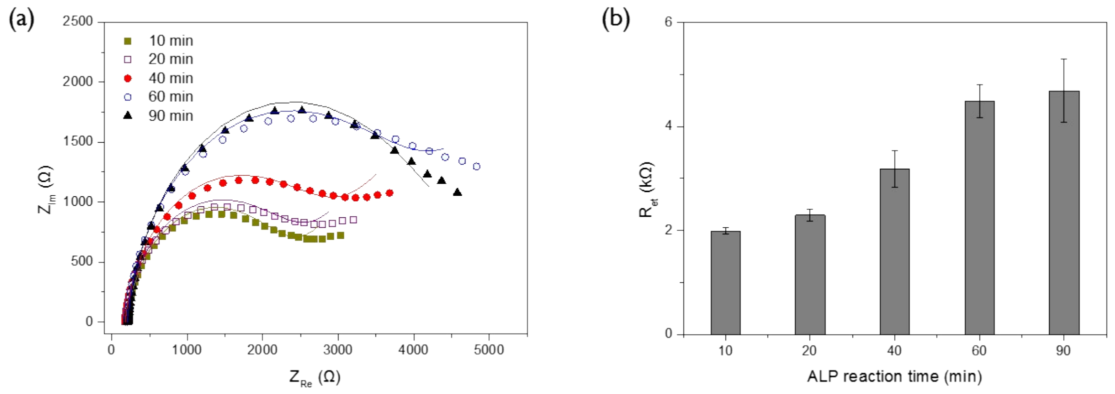

Fig. S2 Optimization of the reaction time for ALP reaction. (a) Nyquist plots of the impedance spectra and (b) electron transfer resistance (R_{et}) obtained from the corresponding impedance spectra at different ALP reaction times. The final concentrations of ALP, PPi, Cu^{2+}, and ascorbic acid are 1 nM, 100 µM, 100 µM, and 2 mM, respectively.

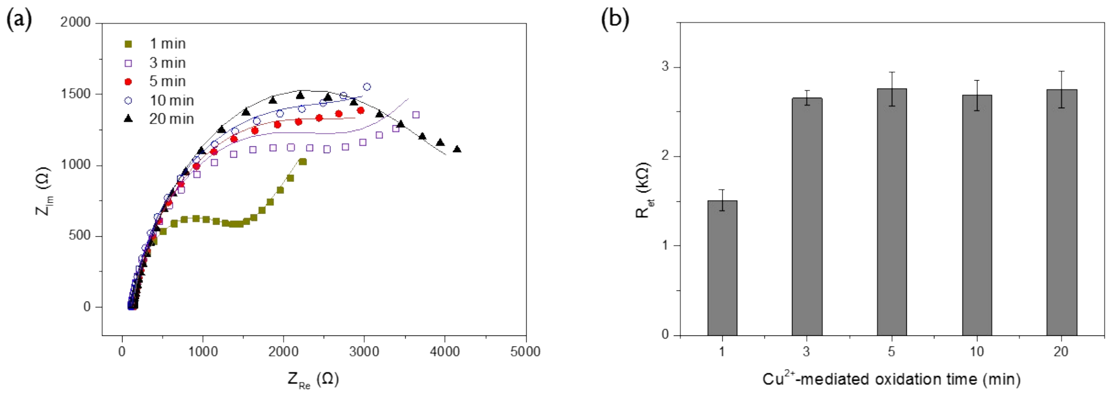

Fig. S3 Optimization of the reaction time for Cu^{2+}-mediated oxidation of ascorbic acid on the poly-thymine DNA-modified electrode. (a) Nyquist plots of the impedance spectra and (b) electron transfer resistance (R_{et}) obtained from the corresponding impedance spectra at different Cu^{2+}-mediated oxidation times. The final concentrations of ALP, PPi, Cu^{2+}, and ascorbic acid are 1 nM, 100 μM, 100 μM, and 2 mM, respectively.

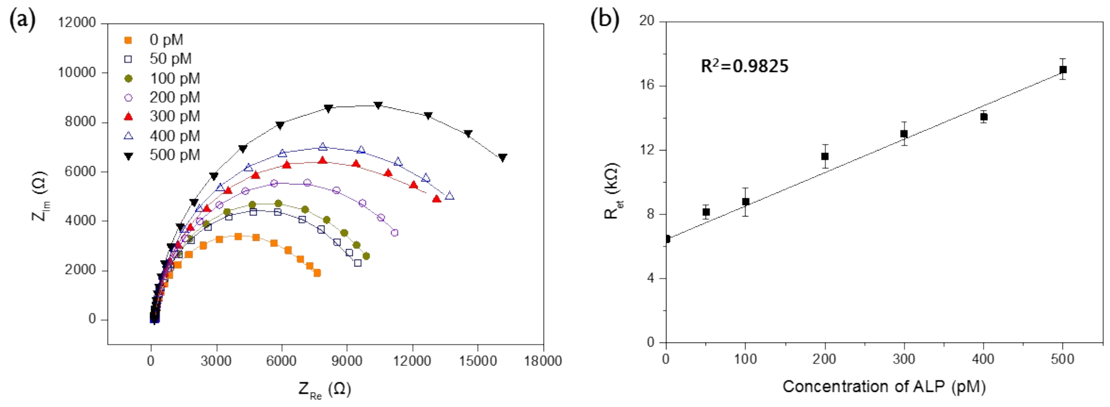

Fig. S4 ALP assay in human serum samples. (a) Nyquist plots of the impedance spectra and (b) electron transfer resistance (R_{et}) obtained from the corresponding impedance spectra upon Cu^{2+}-mediated oxidation in the presence of diluted human serum spiked with varying concentrations of ALP.

Table S1 Comparison of this method with previous electrochemical methods.

Key material/method	Detection limit (U/L)	Linear range (U/L)	Limitations	Reference
Nitrophenylphosphate plastic membrane sensor	30	30 - 3400	- Time-consuming preparation of membrane sensor (28 hr)<br>- Low sensitivity	(Hassan et al. 2009)
λ Exonuclease-mediated signal amplification	100	1000 - 20000	- Requirement of additional enzyme<br>- Low sensitivity	(Miao et al. 2011)
Nanoceria particle-mediated signal amplification	20	5000 - 640000	- Low sensitivity	(Hayat et al. 2013)
Ferrocene-derived substrate	0.4	1 - 250	- Complex synthesis of organic substrate	(Goggins et al. 2015)
Cu^{2+}-mediated oxidation	7.2	22 - 565	-	This work

References

1. S. S. Hassan, H. E. Sayour and A. H. Kamel, *Anal. Chim. Acta*, 2009, **640**, 75-81.

2. P. Miao, L. Ning, X. Li, Y. Shu and G. Li, *Biosens. Bioelectron.*, 2011, **27**, 178-182.

3. A. Hayat and S. Andreescu, *Anal. Chem.*, 2013, **85**, 10028-10032.

4. S. Goggins, C. Naz, B. J. Marsh and C. G. Frost, *Chem. Commun.*, 2015, **51**, 561-564.
